# The time-domain Cartesian multipole expansion of electromagnetic fields

**DOI:** 10.1038/s41598-024-58570-1

**Published:** 2024-04-06

**Authors:** Elias Le Boudec, Chaouki Kasmi, Nicolas Mora, Farhad Rachidi, Emanuela Radici, Marcos Rubinstein, Felix Vega

**Affiliations:** 1 Ecole polytechnique fédérale de Lausanne, Lausanne, Switzerland; 2 Technology Innovation Institute, Abu Dhabi, United Arab Emirates; 3 Universidad Nacional de Colombia, Bogotá, Colombia; 4 Universitá degli Studi dell’Aquila, L’Aquila, Italy; 5 University of Applied Sciences and Arts Western Switzerland, Yverdon-les-Bains, Switzerland

**Keywords:** Electromagnetic radiation, Multipole expansion, Partial differential equations, Maxwell’s equations, Applied mathematics, Electrical and electronic engineering, Physics

## Abstract

Time-domain solutions of Maxwell’s equations in homogeneous and isotropic media are paramount to studying transient or broadband phenomena. However, analytical solutions are generally unavailable for practical applications, while numerical solutions are computationally intensive and require significant memory. Semi-analytical solutions (e.g., series expansion), such as those provided by the current theoretical framework of the multipole expansion, can be discouraging for practical case studies. This paper shows how sophisticated mathematical tools standard in modern physics can be leveraged to find semi-analytical solutions for arbitrary localized time-varying current distributions thanks to the novel time-domain Cartesian multipole expansion. We present the theory, apply it to a concrete application involving the imaging of an intricate current distribution, verify our results with an existing analytical approach, and compare the proposed method to a finite-difference time-domain numerical simulation. Thanks to the concept of current “pixels” introduced in this paper, we derive time-domain semi-analytical solutions of Maxwell’s equations for arbitrary planar geometries.

## Introduction

Solutions of Maxwell’s equations for arbitrary charge and current densities are often impossible to derive analytically, and numerical solutions require long computation times and significant memory. However, semi-analytic closed-form solutions (e.g., solutions involving a series expansion) are sometimes needed to derive fundamental results, allow model fitting, or provide insight into the underlying physics. For example, essential properties of electromagnetic time reversal can be evaluated using time-domain solutions of Maxwell’s equations. This paper presents a systematic approach to derive time-domain semi-analytical expressions for the electromagnetic fields radiated by time-varying localized sources. This time-domain approach is valid for any frequency independently of the source size. The sources are modeled by Schwartz distributions,^1,2^ which offer a generalization of functions to objects such as the Dirac $\delta$ distribution and its derivatives. Locally, distributions can be represented as derivatives of continuous functions.

The multipole expansion is a well-known approach to finding series representations of solutions of Maxwell’s equations. It has been thoroughly studied in the time and frequency domain^3–10^. Some issues involving the definitions of point sources and Green’s functions have been studied within the scope of distribution theory^9,11,12^. In particular, there have been mentions^13–16^ of the link between the spherical and Cartesian multipole expansions and derivatives of the Dirac $\delta$ distribution, including a detailed analysis^17^ of this link in frequency domain. In frequency domain and spherical coordinates, the multipole expansion relies on the separation of variables^18^, decomposing scalar solutions of the Helmholtz equation into a product of angle-dependent functions (the spherical harmonics, i.e., the restriction to the sphere of harmonic and homogeneous polynomials in $\mathbb {R}^3$), and radius-dependent functions (spherical Bessel or Hankel functions or both). As already studied within the framework of pseudopotentials^19^ or distributions in spherical coordinates^20–22^, the singular behavior of the spherical multipole expansion is not trivial. This singular behavior is slightly easier in Cartesian coordinates, as the Cartesian derivatives of the Dirac $\delta$ distribution are well-defined.

This paper builds on the existing approaches involving Schwartz distributions and introduces the time-domain Cartesian multipole expansion. Such an expansion cannot trivially be derived from its frequency-domain counterpart. Indeed, performing a Taylor series expansion of Green’s function in the time domain is formally impossible, as this function is represented by a singular Schwartz distribution. Also, in contrast with the existing literature, we show how to apply the technique to realistic configurations involving an intricate current distribution, thanks to the proposed concept of current pixel. The results are validated by comparing to a finite-difference time-domain numerical simulation and an existing approach^8^. In contrast with the latter reference, our work is based on a different class of time-varying moment, which involves a three- (instead of four-) dimensional integration and offers a straightforward implementation for rectangular domains, such as printed circuit boards. Moreover, it is self-contained in that it can be entirely derived using Schwartz distributions. We also provide an open-source Python implementation of the theory, allowing the reader to experiment with custom current distributions. Finally, the contribution of the charge is explicit, offering a straightforward interpretation at low frequencies.

This paper is organized as follows. First, Section The time-domain Cartesian multipole expansion of electromagnetic fields presents the method, that is, the time-domain Cartesian multipole expansion, and applies it to electromagnetic fields. This method is then illustrated and validated in Section Application to intricate current distributions by computing the electric field radiated by broadband intricate current distributions. Section Discussion comments on the results and the proposed method. Finally, Section Conclusion concludes the paper.

## The time-domain Cartesian multipole expansion of electromagnetic fields

### A generalized Cartesian multipole expansion

We are interested in electromagnetic radiation from localized sources in free space in a homogeneous and isotropic medium described by constant electric permittivity $\varepsilon =\varepsilon _r\varepsilon _0$ and magnetic permeability $\mu =\mu _r\mu _0$.We use the following convention for the partial derivative $\partial _i$, $i=0,1,2,3$: $\partial _0 = \frac{\partial }{\partial t}, \quad \partial _1 = \frac{\partial }{\partial x_1}, \quad \partial _2 = \frac{\partial }{\partial x_2}, \quad \partial _3 = \frac{\partial }{\partial x_3}$, and use the multi-index notation $\alpha =(\alpha _1,\alpha _2,\alpha _3)\in \mathbb {N}^3$, with $|\alpha | = \alpha _1+\alpha _2+\alpha _3$, $\alpha ! = \alpha _1!\alpha _2!\alpha _3!$, $D^\alpha = \partial _1^{\alpha _1}\partial _2^{\alpha _2}\partial _3^{\alpha _3}$, and $\textbf{x}^\alpha = x_1^{\alpha _1}x_2^{\alpha _2}x_3^{\alpha _3}$.

In classical electrodynamics, the radiation of a point charge distribution can be evaluated by computing the Taylor series of Green’s function. Here, we show that this procedure is equivalent to taking linear combinations of derivatives of the Dirac $\delta$ distribution.

Suppose that a scalar distribution *f* satisfies the scalar differential equation

1 $$\begin{aligned} {\left\{ \begin{array}{ll} {\mathcal {L}} f(t,\textbf{x})= \xi (t,\textbf{x})\\ \text {boundary and initial conditions} \end{array}\right. } \end{aligned}$$

where we suppose that the boundary and initial conditions are sufficient for the solution to exist and be unique. Here, ${\mathcal {L}}$ is a linear differential operator with constant coefficients, and $\xi (t,\textbf{x})$ is the source term. For example, for $f=E_1$ (the first electric field component), we have that ${\mathcal {L}}=\Box =\mu \varepsilon \partial _0^2-\nabla ^2$ and $\xi =-1/\varepsilon \partial _1\rho -\mu \partial _0 J_1$. The solution can be represented by a convolution with the appropriate Green’s distribution *G*^23^,

2 $$\begin{aligned} f(t,\textbf{x})=\left[ G(s,\textbf{y})*\xi (s,\textbf{y})\right] (t,\textbf{x}) \end{aligned}$$

where the convolution $*$ is meant over $\mathbb {R}\times \mathbb {R}^3$. Here, we make no particular assumption on Green’s distributions other than its existence and uniqueness. Unfortunately, in general, there are no analytical formulas for such a four-dimensional convolution. Instead, we might replace *G* by some approximation ${\hat{G}}$ with a simpler formulation. If the application $G\mapsto f = G*\xi$ is continuous under some appropriate metric, the fact that ${\hat{G}}$ is close to *G* implies that the approximate solution

3 $$\begin{aligned} {\hat{f}}(t,\textbf{x})=\left[ {\hat{G}}(s,\textbf{y})*\xi (s,\textbf{y})\right] (t,\textbf{x}) \end{aligned}$$

is close to the true solution *f*.

One possible option is to replace *G* with its truncated spatial Taylor series ${\hat{G}}$ of order *n*. In this case, we obtain the classical results for the multipole expansion in Cartesian coordinates. Here, we show that the obtained approximate solution ${\hat{f}}$ can also be derived by approximating the source $\xi$ by a sum of derivatives of Dirac $\delta$ distributions.

#### Theorem 2.1

Let $\Omega \subset \mathbb {R}^3$ be an open set where *G* is spatially analytic and suppose that $\xi$ is a measure with compact support whose spatial moments up to order *n* are finite. Next,

- let ${\tilde{f}}$ be the solution of Eq. (1) when the source term is
  4 $$\begin{aligned} {\tilde{\xi }}(t,\textbf{x})&=\sum _{|\alpha |\le n}\frac{(-1)^{|\alpha |}}{\alpha !}C_\alpha (t)D^\alpha \delta ^3(\textbf{x})\\ &=C_{\textbf{0}}(t)\delta ^3(\textbf{x})-C_{(1,0,0)}(t)\partial _1\delta ^3(\textbf{x})+\cdots +C_{(1,0,1)}(t)\partial _{13}^2\delta ^3(\textbf{x})+\cdots \end{aligned}$$
  where $$C_{\alpha }(t)=\iiint \textbf{y}^\alpha \xi (t, \mathop {}\!\textrm{d}^3\textbf{y})$$,
- and let ${\hat{f}}$ be the multipole expansion in Cartesian coordinates: ${\hat{f}}(t,\textbf{x})=\left[ {\hat{G}}(s,\textbf{y})*\xi (s,\textbf{y})\right] (t,\textbf{x})$.

Then $${\hat{f}}={\tilde{f}} \text { almost everywhere in }\mathbb {R}\times \Omega$$.

The proof of Theorem 2.1 is in the supplementary information. The compact support of $\xi$ means that the source is localized and that we turn the sources on during a finite duration. This hypothesis holds for many systems of interest in physics and engineering. Also, Theorem 2.1 offers a generalization of the multipole expansion approach. Indeed, even when *G* is not analytical, such as with the wave equation in the time domain, it is still possible to convolve it with a point source as in Eq. (4). Moreover, this result is not constrained to electromagnetic fields but applies to any field solution of a differential equation similar to Eq. (1).

### Application to electromagnetic fields

Directly from Maxwell’s equations, we derive the electric and magnetic field wave equations:

5 $$\begin{aligned} \Box \textbf{E}(t,\textbf{x})&= - \frac{1}{\varepsilon } \nabla \rho (t,\textbf{x}) - \mu \partial _0 \textbf{J}(t,\textbf{x}) \end{aligned}$$

6 $$\begin{aligned} \Box \textbf{B}(t,\textbf{x})&= \mu \nabla \times \textbf{J}(t,\textbf{x}) \end{aligned}$$

where $\Box =\mu \varepsilon \partial ^2_0 - \nabla ^2$ is the d’Alembert operator. It is interesting to start from Eqs. (5), (6), as Green’s distribution is known to be^23,24^ $\pm 1/(4\pi )\delta (t\mp |\textbf{x}|/c)/|\textbf{x}|$ (the sign determines causality). Also, the electric and magnetic fields are seemingly decoupled. Of course, this coupling exists, and it is embedded in the continuity equation:

7 $$\begin{aligned} 0 = \partial _0\rho (t,\textbf{x})+\nabla \cdot \textbf{J}(t,\textbf{x}) \end{aligned}$$

Now, notice that the *i*th component ($i=1,2,3$) of the wave equation for the electric field (Eq. 5) can be written as

8 $$\begin{aligned} \Box E_i(t,\textbf{x})=-\frac{1}{\varepsilon }\partial _i \rho (t,\textbf{x})-\mu \partial _0 J_i(t,\textbf{x}) \end{aligned}$$

Let us focus on the right-hand side. By performing a multipole expansion in Cartesian coordinates and by including the continuity equation, both the current and the charge densities consist of sums of derivatives of Dirac $\delta ^3$ distributions, where each term of the sum may be symbolically written as $C_\alpha (t)D^{{\alpha }} \delta ^3(\textbf{x})$ for some appropriate time-dependent function $C_\alpha$ and some appropriate derivative $D^{{\alpha }}$. This type of distribution models a time-dependent point source. Explicitly, the distribution $C_\alpha (t) D^{\alpha }\delta ^3(\textbf{x})$ acts on any test function $\psi \in {\mathcal {D}}$ as

9 $$\begin{aligned} \left\langle C_\alpha (t) D^{\alpha }\delta ^3(\textbf{x}),\psi (t,\textbf{x})\right\rangle = (-1)^{|\alpha |}\int _{\mathbb {R}} C_\alpha (t)(D^{\alpha }\psi )(t,\textbf{0})\mathop {}\!\textrm{d}t \end{aligned}$$

by definition of the derivative of a distribution, and where ${\mathcal {D}}$ denotes the set of compactly-supported smooth functions mapping space-time coordinates to real numbers. In Eq. (9), we can treat the time variable *t* independently from the space variable $\textbf{x}$. Since $C_\alpha$ is a regular distribution, the effect of $C_\alpha$ on $\psi$ is a time integral. On the other hand, the Dirac $\delta$ distribution evaluates (the derivative of) $\psi$ at $\textbf{x}=\textbf{0}$, which is why the integrand in Eq. (9) is $(-1)^{|\alpha |} C_\alpha (t)(D^\alpha \psi )(t,\textbf{0})$. In light of the above, the right-hand side of Eq. (8) can be written as a finite sum of time-dependent point sources, say $\Box E_i(t,\textbf{x})=\sum _{j=1}^{n}C_j(t)D^{\alpha _j}\delta ^3(\textbf{x})$ for some integer *n*.

Now, suppose that we know a solution to the simpler differential equation $\Box E_i^j(t,\textbf{x})= C_j(t)D^{\alpha _j}\delta ^3(\textbf{x})$. Then, by the linearity of the wave equation and since the initial conditions are zero for $t<0$, the field $E_i(t,\textbf{x})=\sum _{j=1}^{n}E_i^j(t,\textbf{x})$ is a solution of the original differential equation for the *i*th component of the electric field, namely, $\Box E_i(t,\textbf{x})= \sum _{j=1}^{n} C_j(t)D^{\alpha _j}\delta ^3(\textbf{x})$, with $\alpha \in \mathbb {N}^3$. Of course, the same applies to the magnetic field.

By this reasoning, the solution of the electromagnetic wave equations with time-dependent point source densities can be determined by the generic solution $f_\alpha (t,\textbf{x};C)$ of the scalar differential equation

10 $$\begin{aligned} \Box f_\alpha (t,\textbf{x};C) = C(t) D^\alpha \delta ^3(\textbf{x}) \end{aligned}$$

where the function *C* describes the time dependence. Next, we will show that the solution of Eq. (10) can be computed recursively. To this end, let us define the auxiliary function $g_{\alpha }(t,\textbf{x};C)$ as

- If $$\alpha =\textbf{0}:$$
  11 $$\begin{aligned} g_{\textbf{0}}(t,\textbf{x};C)=\pm \frac{C(t)}{4\pi |\textbf{x}|} \end{aligned}$$
  The signs correspond to causal ($+$) and anti-causal (−) solutions.
- Else, for $|\alpha |>0$, let *j* be the first nonzero dimension of $\alpha$, $c^2=(\mu \varepsilon )^{-1}$, and we have the recursion relation
  recursion relation $$\begin{aligned} g_{\alpha }(t,\textbf{x};C)= (\partial _j g_{\alpha -\textbf{e}_j}) (t,\textbf{x};C)\mp (\partial _0g_{\alpha -\textbf{e}_j})(t,\textbf{x};C)\frac{x_j}{c|\textbf{x}|} \end{aligned}$$
  As above, the signs correspond to causal (−) and anti-causal ($+$) solutions.

As an example, the causal recursion relation for $\alpha =(1,0,0)$ reads

12 $$\begin{aligned} g_{(1,0,0)}(t,\textbf{x};C)= (\partial _1 g_{\textbf{0}})(t,\textbf{x};C)-(\partial _0g_{\textbf{0}}) (t,\textbf{x};C)\frac{x_1}{c|\textbf{x}|}=-\frac{x_1}{4\pi }\left[ \frac{C(t)}{|\textbf{x}|^3} +\frac{\mathop {}\!\textrm{d}C(t)}{\mathop {}\!\textrm{d}t}\frac{ 1}{c|\textbf{x}|^2}\right] \end{aligned}$$

We can explicitly compute the expression of $g_\alpha (t,\textbf{x}; C)$ using the recursion relation with any symbolic programming language. Note that $g_{\alpha }$ is a real-valued function defined on $\mathbb {R}\times (\mathbb {R}^3\backslash \{0\})$. It depends only on the space coordinate $\textbf{x}$ and the derivatives of the time-dependence *C*. Also, it always contains a term in $1/|\textbf{x}|$, linked to a time derivative of order $|\alpha |$.

We are now ready to solve Eq. (10):

#### Theorem 2.2

Let $f_\alpha$ be a solution of the scalar differential Eq. (10). Then

$$\begin{aligned} \left\langle f_\alpha (t,\textbf{x};C),\psi (t,\textbf{x})\right\rangle =\left\langle g_{\alpha } (t\mp |\textbf{x}|/c,\textbf{x};C),\psi (t,\textbf{x})\right\rangle \end{aligned}$$

for every smooth $\psi$ with compact support such that its derivatives of any order vanish at $\textbf{x}=\textbf{0}$. This implies that $g_\alpha (t\mp |\textbf{x}|/c,\textbf{x}; C)$ can be considered in a suitable weak sense as a solution of Eq. (10).

The weaker statement chosen in the theorem allows to circumvent subtleties around the singular behavior of the solution at the origin. The proof of Theorem 2.2 is in the supplementary information.

## Application to intricate current distributions

In this Section, we apply the aforementioned method to the electric field radiated by intricate current distributions. To simplify the derivation, we assume that the time dependence of the current density is separable, i.e., $\textbf{J}(t,\textbf{x})=h(t)\textbf{J}(\textbf{x})$. If this assumption does not hold, the only different result in what follows is the formula for the moments in Eqs. (13) and (14). In both cases, we first determine the charge density $\rho$, given by Eq. (7). By the separability assumption, the charge can be written as $\rho (t,\textbf{x})=\partial _0^{-1}h(t)\rho (\textbf{x})$ where $\partial _0^{-1}h(t)$ is an antiderivative of *h*. Second, we compute the time-domain moments corresponding to the right-hand side of Eq. (8). These moments can be separated into current moments

13 $$\begin{aligned} C_\alpha ^{J_i}(t)=-\mu h'(t)\iiint _{\mathbb {R}^{3}}J_i(\textbf{y})\textbf{y}^\alpha \mathop {}\!\textrm{d}^3\textbf{y}\end{aligned}$$

and charge moments

14 $$\begin{aligned} C_\alpha ^{\rho ,i}(t)=-\frac{1}{\varepsilon }\partial _0^{-1}h(t) \iiint _{\mathbb {R}^{3}}\partial _i\rho (\textbf{y})\textbf{y}^\alpha \mathop {}\!\textrm{d}^3\textbf{y}\end{aligned}$$

Third, we recursively compute the auxiliary function $g_\alpha$ up to the desired order *n*, thanks to the recursion relation. Finally, by Theorem 2.2, the components of the electric field are given by

15 $$\begin{aligned} E_i(t,\textbf{x})=\sum _{|\alpha |\le n}g_\alpha \left( t-|\textbf{x}|/c,\textbf{x};C_ \alpha ^{J_i}(t)+C_\alpha ^{\rho ,i}(t)\right) \end{aligned}$$

Similar to how on-screen pixels represent smooth curves by concatenations of rectangles, it is reasonable to assume that any planar current density can be decomposed into an appropriately large set of rectangular current “pixels”, with a negligible effect on the radiated field. To find such a decomposition, we first determine the radiation of such a pixel, aligned to the $x_1$- and $x_2$-axes, centered at the coordinates $(x_1^0,x_2^0,0)$, of width $\sigma _1$ and height $\sigma _2$. We suppose that a time-varying current density *h* travels across it in the direction of $\textbf{e}_1$. Such a current distribution corresponds to

16 $$\begin{aligned} \textbf{J}(t,\textbf{x}) =h(t)\delta (x_3)\Pi \left( \frac{x_1-x_1^0}{\sigma _1}\right) \Pi \left( \frac{x_2-x_2^0}{\sigma _2}\right) \textbf{e}_1 \end{aligned}$$

where $\Pi$ is the Heaviside $\Pi$ function, equal to 1 for an argument between $-\frac{1}{2}$ and $\frac{1}{2}$, and zero otherwise. To simplify the derivation, we focus on the first component of the electric field, which is the most difficult since the current is polarized along the first axis. Of course, what follows can be generalized to the other components of the electric or magnetic fields. Equation (8) shows that we also need to compute the derivative of the charge density, which we express as a function of the current density thanks to the continuity equation, Eq. (7):

17 $$\begin{aligned} \partial _1\rho (t,\textbf{x})=-\partial _1\partial _0^{-1} \partial _1J_1(t,\textbf{x}) =-\partial ^{-1}_0h(t)\delta (x_3) \partial _1^2\left[ \Pi \left( \frac{x_1-x_1^0}{\sigma _1}\right) \right] \Pi \left( \frac{x_2-x_2^0}{\sigma _2}\right) \end{aligned}$$

A detailed computation of the moments is presented in the supplementary information. Next, any intricate current distributions on the $x_1x_2$-plane, polarized along the first axis, can be decomposed into a sum of adjacent and disjoint rectangular pixels: $\textbf{J}(t,\textbf{x})=\sum _{i=1}^{m}\textbf{J}^{(i)}(t,\textbf{x})$ where each rectangular current pixel $\textbf{J}^{(i)}$ can be expressed according to Eq. (16). By linearity of the wave equation, the total field is given by the sum of the contribution of every current pixel. Note that this decomposition introduces charge accumulation at the current pixels’ boundaries. Nevertheless, this introduction is not spurious: on the one hand, if all current pixels have the same time dependence *h*, as in the examples below, then the neighboring charges cancel each other since they have the same amplitude (depending on *h*), but opposite polarities. On the other hand, if we admit varying time-dependencies $h^{(i)}$ for every pixel *i*, then these boundary charges do not cancel, reflecting the non-zero divergence of the current density in that case.

**Figure 1:**
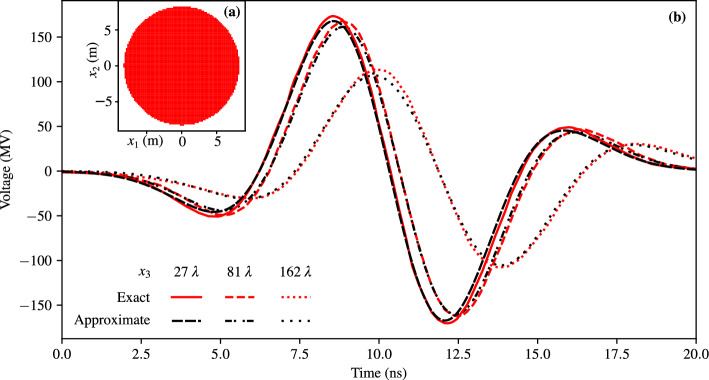
Comparison of the semi-analytical result with the literature^8^. **(a)** Distribution of the current density approximating a disc across the $x_1x_2$-plane. The disc is formed of 5744 square current pixels. **(b)** The voltage $x_3 E_1$ corresponding to the component of the electric field radiated by a disc at the points $\textbf{x}=(0,0,27\lambda )$, $\textbf{x}=(0,0,81\lambda )$ and $\textbf{x}=(0,0,162\lambda )$. We compare the 24th-order Cartesian multipole expansion of the approximate disc with the spherical multipole expansion of the exact disc^8^.

**Figure 2:**
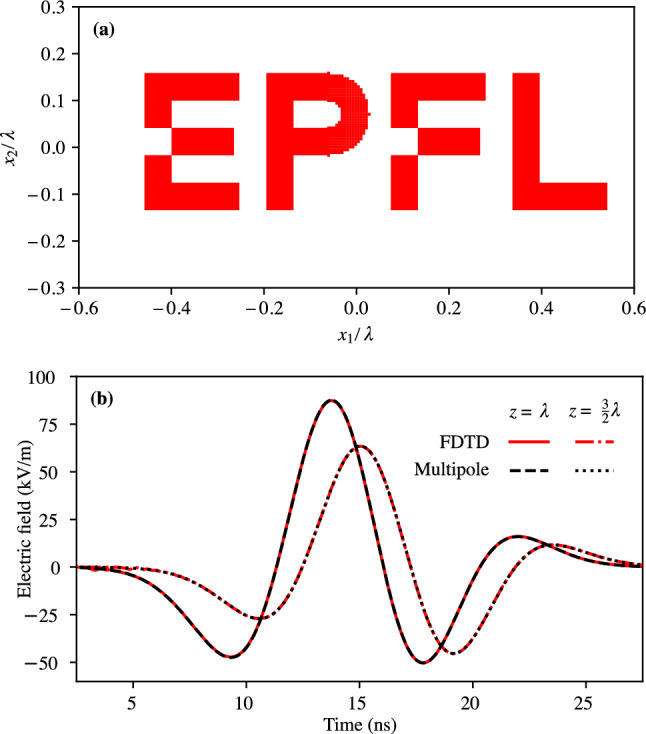
Comparison of the proposed method to a finite-difference time-domain (FDTD) numerical simulation. (**a**) The current density across the $x_1x_2$-plane for the intricate current distribution. The curvy part of the “P” is approximated by over 300 square current pixels. (**b**) The first component of the electric field radiated by the intricate current density at the points $\textbf{x}=(0,0,\lambda )$ and $\textbf{x}=(0,0,3/2\lambda )$. We compare an 8th-order multipole expansion with an FDTD numerical simulation.

**Figure 3:**
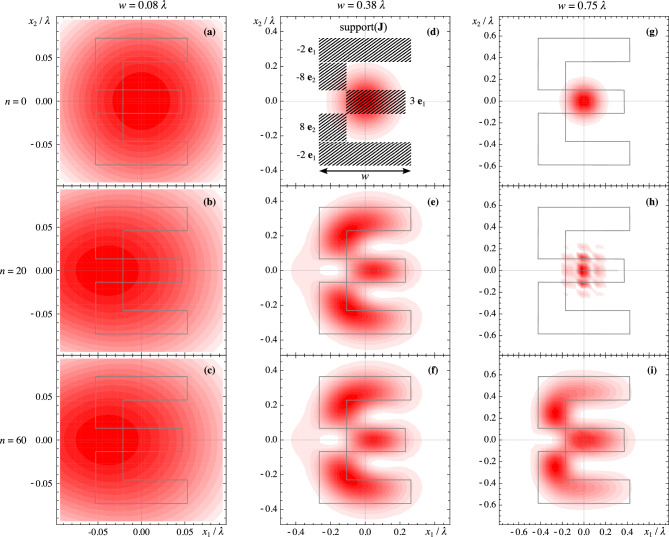
Normalized norm of the electric field obtained from the imaging experiment at $t=0$ and $x_3=0$. Three electrical sizes are illustrated along the three columns. The rows indicate the truncation order *n*. The current density, whose outline is illustrated, is obtained by splitting the letter “E” into five rectangles whose amplitudes and polarities are also indicated in (**d**).

To illustrate and validate the presented approach, we present three case studies:

1. First, we validate the method by computing the electric field radiated by a broadband Gaussian pulse^8^ of length $T=3.06\textrm{ns}$ and smallest wavelength $\lambda =cT$, flowing through the geometry illustrated in Fig. 1a (a disc of radius $9\lambda$). We then compare the obtained approximate result to the alternative method^8^, which uses a multipole expansion in spherical coordinates and a different class of time-domain moments.
2. Second, we compute the electric field radiated by a broadband Gaussian pulse (as above) flowing through the intricate geometry illustrated in Fig. 2a. We compare the near-field obtained semi-analytically by computation of the moments with a finite-difference time-domain (FDTD) numerical simulation obtained using Meep^25^. The side length of the current distribution corresponds to $\lambda$; thus, the source is neither electrically large (much bigger than $\lambda$) nor electrically small (much smaller than $\lambda$). We probe the field at a distance equal to $\lambda$ and $3/2 \lambda$.
3. Third, we illustrate the method in an imaging experiment. We consider an intricate current density forming the letter “E” carrying the pulse $h(t)=e^{-(t/T)^2}$, where *T* is such that the 3 dB cutoff frequency corresponds to the wavelength $\lambda$. The imaging experiment is obtained by superposing the causal and anti-causal solutions (see Eq. (15))
  18 $$\begin{aligned} E_i(t,\textbf{x})=\sum _{|\alpha |\le n}g_\alpha \left( t-|\textbf{x}|/c,\textbf{x};C_\alpha ^{J_i}(t)+C_\alpha ^{\rho ,i}(t)\right) -g_\alpha \left( t+|\textbf{x}|/c,\textbf{x};C_\alpha ^{J_i}(t)+C_\alpha ^{\rho ,i}(t)\right) \end{aligned}$$
  and analyzing the electric field at $t= 0s$. This corresponds to an ideal time-reversal cavity^26^. The current density and corresponding fields are illustrated in Fig. 3.

In all three presented cases, the current density is assumed to be two-dimensional for simplicity of illustration and comparison. However, the corresponding radiation problem is fully three-dimensional, as is the presented method. The results are discussed in the next section.

## Discussion

**Figure 4:**
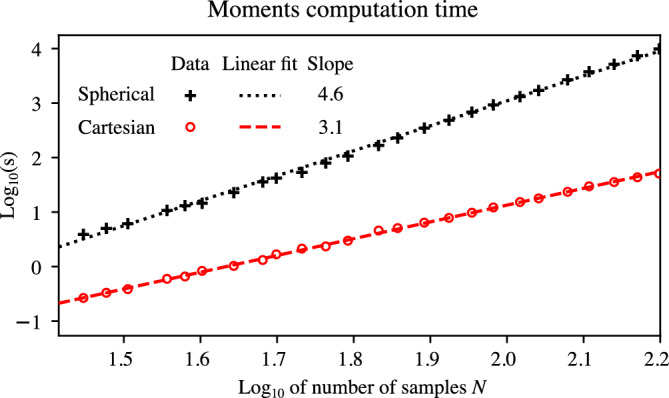
Computation time of the spherical and Cartesian moments as a function of the number of moment samples *N*.

In the first case, illustrated in Figure 1, as expected, we see that the semi-analytical method proposed here matches the similar approach presented in the literature^8^. Compared to the latter reference, the proposed time-domain moments rely on a three-dimensional integral, whereas the existing method needs a four-dimensional integral. Having one less integral to perform simplifies numerical implementations. Figure 4 presents a comparison of the computation time (averaged over three samples) of the spherical moment $l=2$, $m=1$ and the corresponding Cartesian moment $\alpha =(1,0,1)$ (p. 287 in^27^) for the first case study. The moments are computed from a smooth approximation of the current density, and the integral is discretized as a sum. The spatial samples are evenly spaced and their number is *N* per dimension. For the spherical moments, the discretization of $\xi$ in eqs. (22a-b) of^8^ is set to 2*N* evenly spaced samples. The computation is randomly distributed over eight processes of an Apple M1 Max computer.

As expected, the Cartesian moments are significantly faster to compute, having a cubic complexity compared to the quartic-to-quintic complexity of the spherical moments (see the slopes in Figure 4).

The next difference between both methods lies in using the Cartesian multipole expansion versus the spherical multipole expansion. Theoretically, both methods are comparable, although the singular behavior of the Cartesian multipole expansion is free from the subtleties appearing when using spherical coordinates^19,28^. If the geometry can be described by rectangles (such as current pixels), the Cartesian multipole moments are easier to compute. Moreover, the Cartesian formulation enables the computation of the radiation of intricate current distributions described by square current pixels of arbitrary polarization.

In the second case, which compares the proposed approach to a numerical method, we notice in Fig. 2b that the semi-analytical result using an 8th-order multipole expansion matches the numerical simulation results. This shows that, as expected, the method is valid in the near-field for a broadband pulse, where the concept of a single frequency and single wavelength is not applicable. Compared to numerical methods, the proposed approach has several advantages. First, the memory requirement and computation time depend only on the number of observation points. In contrast, FDTD-based simulations are constrained by the size of the domain and the smallest feature in the domain. Finite-element approaches also depend on the required mesh and domain sizes, even for a single observation point. The FDTD simulation of the geometry in Fig. 2a required 676 MiB of memory and took 68 s on an Apple M1 Max computer, compared to less than a second and a MiB for the multipole expansion on the same machine. Second, the result can be displayed in a human-readable series expansion (a semi-analytical result), allowing for finer interpretation and predictions. Finally, the primary constraint for these multipole expansion approaches is the electrical size of the source^8^. Indeed, even in the far field, the expansion order is proportional to the electrical size of the source (ratio of the source diameter and the smallest significant wavelength).

The illustration case study in Fig. 3 shows how the proposed method can be used, together with time-reversal^26^, to predict imaging experiment results. For electrically small sources ($w=0.08\lambda$, Fig. 3a–c), low truncation orders *n* are enough to represent the field accurately (e.g., Fig. 3b). However, the current distribution geometry is lost in the radiated field. By increasing the electrical size (up to $w=0.75\lambda$, Fig. 3d–i), details such as sharp corners become visible (Fig. 3i), and the letter becomes legible (Fig. 3e,f,i). As a tradeoff, the truncation order needs to be increased to $n=60$; below, the truncated field is invalid (Fig. 3a,d,g,h).

## Conclusion

We presented the time-domain Cartesian multipole expansion, a method to derive the electromagnetic fields radiated by any localized time-varying current density in homogeneous and isotropic media. The technique is self-contained thanks to Schwartz distributions and easily implemented in any programming language thanks to its formulation in Cartesian coordinates. Moreover, to the authors’ best knowledge, it is the first explicit time-domain approach in Cartesian coordinates. Moreover, we showed how to apply the method to intricate current densities and validated the result by comparing it to the spherical time-domain multipole expansion and a numerical simulation.

Contrary to numerical methods, once an expression for the multipole expansion is obtained, it can be evaluated efficiently for varying parameters (e.g., the amplitude of the moments, the shape of the time-domain excitation *h*, the origin of the multipole expansion, etc.), opening the door to physics-informed machine learning approaches for model identification. Other applications of interest include problems involving wideband radiation, such as impulse radiating antennas or electrostatic discharges.

### Supplementary Information

Supplementary Information.

## Data Availability

The datasets generated and analyzed during the current study are available in the Pynoza repository, https://doi.org/10.5281/zenodo.10498065.
