# Supplementary material for: The time-domain Cartesian multipole expansion of electromagnetic fields
Source: Sci Rep. 2024 Apr 6;14:8084. doi: 10.1038/s41598-024-58570-1 (PMC10998848; doi:10.1038/s41598-024-58570-1)
Supplement: Supplementary file 1 — Supplementary Information. [file 41598_2024_58570_MOESM1_ESM.pdf]

Supplementary information

Elias Le Boudec[1,*], Chaouki Kasmi[2], Nicolas Mora[3], Farhad Rachidi[1], Emanuela Radici[4], Marcos Rubinstein[5], and Felix Vega[2]

[1]Ecole polytechnique fédérale de Lausanne, Lausanne, Switzerland
[2]Technology Innovation Institute, Abu Dhabi, United Arab Emirates
[3]Universidad Nacional de Colombia, Bogotá, Colombia
[4]Università degli Studi dell'Aquila, L'Aquila, Italy
[5]University of Applied Sciences and Arts Western Switzerland, Yverdon-les-Bains, Switzerland
[*]elias.leboudec@epfl.ch

A Proof of Theorem 2.2

Proof. By the integrability assumption on ξ and since the Taylor series of G exists, we have that

$$\hat{f}(t,\mathbf{x}) = \int_0^T \iiint_{B(\mathbf{0},R)} \hat{G}(t-s,\mathbf{x}-\mathbf{y})\xi(s,\mathrm{d}^3\mathbf{y})\,\mathrm{d}s \tag{1}$$

where the support of ξ is contained in the time interval $[0,T]$ and the ball $B(\mathbf{0},R)$. Next, the Taylor series of $G(t-s,\mathbf{x}-\mathbf{y})$ around $\mathbf{y}=\mathbf{0}$ reads

$$\hat{G}(t-s,\mathbf{x}-\mathbf{y}) = \sum_{|\alpha|\le n} \frac{(-1)^{|\alpha|}}{\alpha!}(D^\alpha G)(t-s,\mathbf{x})\mathbf{y}^\alpha \tag{2}$$

The term $(-1)^{|\alpha|}$ appears because we differentiate with respect to $\mathbf{y}$ but express the Taylor series as a function of the derivatives of G, $(D^\alpha G)$. Therefore, by the linearity of the integral in Equation (1),

$$\hat{f}(t,\mathbf{x}) = \int_0^T \iiint_{B(\mathbf{0},R)} \hat{G}(t-s,\mathbf{x}-\mathbf{y})\xi(s,\mathrm{d}^3\mathbf{y})\,\mathrm{d}s = \int_0^T \iiint_{B(\mathbf{0},R)} \sum_{|\alpha|\le n} \frac{(-1)^{|\alpha|}}{\alpha!}(D^\alpha G)(t-s,\mathbf{x})\mathbf{y}^\alpha \xi(s,\mathrm{d}^3\mathbf{y})\,\mathrm{d}s \tag{3}$$

$$= \int_0^T \sum_{|\alpha|\le n} \frac{(-1)^{|\alpha|}}{\alpha!}(D^\alpha G)(t-s,\mathbf{x}) \iiint_{B(\mathbf{0},R)} \mathbf{y}^\alpha \xi(s,\mathrm{d}^3\mathbf{y})\,\mathrm{d}s = \int_0^T \sum_{|\alpha|\le n} \frac{(-1)^{|\alpha|}}{\alpha!}(D^\alpha G)(t-s,\mathbf{x})C_\alpha(s)\,\mathrm{d}s \tag{4}$$

where we interchanged integration and sum and recognized the definition of C_α.

On the other hand, let us compute the field $\tilde{f}$ radiated by the source $\tilde{\xi}$. To this end, let ψ be a test function whose support is in $\mathbb{R}\times\Omega$. Hence

$$\left\langle \tilde{f}(t,\mathbf{x}), \psi(t,\mathbf{x}) \right\rangle = \left\langle \left[G(s,\mathbf{y}) * \tilde{\xi}(s,\mathbf{y})\right](t,\mathbf{x}), \psi(t,\mathbf{x}) \right\rangle = \left\langle G(t,\mathbf{x}), \left\langle \tilde{\xi}(s,\mathbf{y}), \psi(t+s,\mathbf{x}+\mathbf{y}) \right\rangle \right\rangle \tag{5}$$

(by definition of the convolution, see [1, § 6.2])

$$= \sum_{|\alpha|\le n} \frac{(-1)^{|\alpha|}}{\alpha!} \left\langle G(t,\mathbf{x}), \left\langle C_\alpha(s)(D^\alpha\delta^3)(\mathbf{y}), \quad \psi(t+s,\mathbf{x}+\mathbf{y})\right\rangle\right\rangle = \sum_{|\alpha|\le n} \frac{1}{\alpha!} \left\langle G(t,\mathbf{x}), \left\langle C_\alpha(s)\delta^3(\mathbf{y}), (D^\alpha\psi)(t+s,\mathbf{x}+\mathbf{y})\right\rangle\right\rangle \tag{6}$$

(by definition of $\tilde{\xi}$ and linearity of the convolution product, then by definition of the derivative of a distribution)

$$= \sum_{|\alpha|\le n} \frac{1}{\alpha!} \left\langle G(t,\mathbf{x}), \int_0^T C_\alpha(s)(D^\alpha\psi)(t+s,\mathbf{x})\,\mathrm{d}s \right\rangle \tag{7}$$

by the regularity of $C_\alpha(s)$ and by definition of the Dirac δ distribution. Next, since G is a regular distribution, we get

$$= \sum_{|\alpha|\le n} \frac{1}{\alpha!} \int_{\mathbb{R}} \int_0^T \iiint_{\mathbb{R}^3} G(t,\mathbf{x})C_\alpha(s)(D^\alpha\psi)(t+s,\mathbf{x})\,\mathrm{d}^3\mathbf{x}\,\mathrm{d}s\,\mathrm{d}t \tag{8}$$

$$= \sum_{|\alpha|\le n} \frac{(-1)^{|\alpha|}}{\alpha!} \int_{\mathbb{R}} \int_0^T \iiint_{\mathbb{R}^3} (D^\alpha G)(t,\mathbf{x}) C_\alpha(s) \psi(t+s,\mathbf{x})\, \mathrm{d}^3\mathbf{x}\, \mathrm{d}s\, \mathrm{d}t \tag{9}$$

by integration by parts and the compact support of ψ. Now, we introduce the change of coordinates $\begin{bmatrix} t & s & \mathbf{x} \end{bmatrix} \mapsto \begin{bmatrix} u & s & \mathbf{x} \end{bmatrix} = \begin{bmatrix} t+s & s & \mathbf{x} \end{bmatrix}$. The determinant of the Jacobian matrix of this change of coordinates is 1. We thus obtain

$$= \sum_{|\alpha|\le n} \frac{(-1)^{|\alpha|}}{\alpha!} \int_{\mathbb{R}} \int_0^T \iiint_{\mathbb{R}^3} (D^\alpha G)(u-s,\mathbf{x}) C_\alpha(s) \psi(u,\mathbf{x})\, \mathrm{d}^3\mathbf{x}\, \mathrm{d}s\, \mathrm{d}u \tag{10}$$

Here, we recognize the expression of the regular distribution

$$\tilde{f}(t,\mathbf{x}) = \int_0^T \sum_{|\alpha|\le n} \frac{(-1)^{|\alpha|}}{\alpha!} (D^\alpha G)(t-s,\mathbf{x}) C_\alpha(s)\, \mathrm{d}s \tag{11}$$

Comparing with Equation (4), it is clear that $\langle \hat{f}, \psi \rangle = \langle \tilde{f}, \psi \rangle$ for all test functions ψ such that support$(\psi) \subset \mathbb{R}\times\Omega$. By [1, Theorem 6.4], this means $\hat{f} = \tilde{f}$ almost everywhere in $\mathbb{R}\times\Omega$. □

B Proof of Theorem 2.2

Proof. We will show the result by recursion on the derivative order $|\alpha|$. First, we show the result for $|\alpha| = 0$, i.e., $\alpha = \mathbf{0}$. Second, even though it is not formally needed, we also show the result for $|\alpha| = 1$. We show this case because it introduces almost all the mechanisms we need for the recursion step while keeping a relatively short notation. We finally show the recursion step.

The goal of the proof is to show that for any test function $\psi \in \mathscr{D}$ whose derivatives vanish at $\mathbf{x} = \mathbf{0}$,

$$\langle f_\alpha(t,\mathbf{x};C), \psi(t,\mathbf{x}) \rangle = \langle g_\alpha(t \mp |\mathbf{x}|/c, \mathbf{x}; C), \psi(t,\mathbf{x}) \rangle \tag{12}$$

The requirement $D^\alpha \psi(t,\mathbf{0}) = 0$ for any α comes from the singularity of g_α at $\mathbf{x} = \mathbf{0}$. It ensures that all integrals below are well-defined. With this in mind, let us proceed to the proof of the recursion relation, as described above.

First, let us show the result for $\alpha = \mathbf{0}$. We need to find $f_\mathbf{0}$ satisfying $\Box f_\mathbf{0}(t,\mathbf{x};C) = C(t)\delta^3(\mathbf{x})$. To this end, we use the fact[2,3] that the corresponding Green's distribution is uniquely determined by $G(t,\mathbf{x}) = \pm\frac{1}{4\pi}\frac{\delta(t\mp|\mathbf{x}|/c)}{|\mathbf{x}|}$. Hence, f is given by the convolution product $G(s,\mathbf{y}) * [C(s)\delta^3(\mathbf{y})]$. Since neither Green's distribution nor the source term $C(s)\delta^3(\mathbf{y})$ is regular, we must use the most general definition of the convolution product [1, Section 6.2], which reads, for any test function $\psi \in \mathscr{D}$ whose derivatives vanish at $\mathbf{x} = \mathbf{0}$,

$$\langle f_\mathbf{0}(t,\mathbf{x};C), \psi(t,\mathbf{x}) \rangle = \left\langle [G(s,\mathbf{y}) * (C(s)\delta^3(\mathbf{y}))](t,\mathbf{x}), \psi(t,\mathbf{x}) \right\rangle = \left\langle G(t,\mathbf{x}), \left\langle C(s)\delta^3(\mathbf{y}), \psi(t+s,\mathbf{x}+\mathbf{y}) \right\rangle \right\rangle \tag{13}$$

(by [1, 6.2])

$$= \left\langle G(t,\mathbf{x}), \int_{\mathbb{R}} C(s)\psi(t+s,\mathbf{x})\, \mathrm{d}s \right\rangle = \left\langle \frac{\pm 1}{4\pi} \frac{\delta(t\mp|\mathbf{x}|/c)}{|\mathbf{x}|}, \int_{\mathbb{R}} C(s)\psi(t+s,\mathbf{x})\, \mathrm{d}s \right\rangle \tag{14}$$

(by Equation (9) of the main text and the definition of G)

$$= \iiint_{\mathbb{R}^3} \int_{\mathbb{R}} \frac{\pm 1}{4\pi} \frac{C(s)}{|\mathbf{x}|} \psi(s \pm |\mathbf{x}|/c, \mathbf{x})\, \mathrm{d}s\, \mathrm{d}^3\mathbf{x} \tag{15}$$

Now, we make the change of coordinates

$$\begin{bmatrix} s & \mathbf{x} \end{bmatrix} \mapsto \begin{bmatrix} t & \mathbf{x} \end{bmatrix} = \begin{bmatrix} s \pm |\mathbf{x}|/c & \mathbf{x} \end{bmatrix} \tag{16}$$

Since the determinant of the Jacobian matrix of this change of coordinates is 1, we get

$$\langle f_\mathbf{0}(t,\mathbf{x};C), \psi(t,\mathbf{x}) \rangle = \iiint_{\mathbb{R}^3} \int_{\mathbb{R}} \frac{\pm 1}{4\pi} \frac{C(t\mp|\mathbf{x}|/c)}{|\mathbf{x}|} \psi(t,\mathbf{x})\, \mathrm{d}t\, \mathrm{d}^3\mathbf{x} \tag{17}$$

At this point, we recognize the expression of the regular distribution

$$f_\mathbf{0}(t,\mathbf{x};C) = \frac{\pm 1}{4\pi} \frac{C(t\mp|\mathbf{x}|/c)}{|\mathbf{x}|} = g_\mathbf{0}(t \mp |\mathbf{x}|/c, \mathbf{x}; C) \tag{18}$$

Second, let us show the result for $|\alpha| = 1$. Let $\alpha = (1,0,0)$ and ψ be as before. We proceed as for $\alpha = \mathbf{0}$:

$$\langle f_\alpha(t,\mathbf{x};C), \psi(t,\mathbf{x})\rangle = \left\langle G(t,\mathbf{x}), \left\langle C(s)D^{(1,0,0)}\delta^3(y), \psi(t+s,\mathbf{x}+\mathbf{y})\right\rangle\right\rangle$$
$$= \langle G(t,\mathbf{x}), \langle C(s)\partial_1\delta^3(\mathbf{y}), \psi(t+s,\mathbf{x}+\mathbf{y})\rangle\rangle = -\langle G(t,\mathbf{x}), \langle C(s)\delta^3(\mathbf{y}), (\partial_1\psi)(t+s,\mathbf{x}+\mathbf{y})\rangle\rangle \quad (19)$$

by definition of the derivative of a distribution. Using equations (13) to (15),

$$= -\iiint_{\mathbb{R}^3}\int_{\mathbb{R}} g_{\mathbf{0}}(s,\mathbf{x};C)(\partial_1\psi)(s\pm|\mathbf{x}|/c,\mathbf{x})\,\mathrm{d}s\,\mathrm{d}^3\mathbf{x} \quad (20)$$

Now, we use the following result from differentiation:

$$\partial_1[\psi(s\pm|\mathbf{x}|/c,\mathbf{x})] = (\partial_1\psi)(s\pm|\mathbf{x}|/c,\mathbf{x}) \pm (\partial_0\psi)(s\pm|\mathbf{x}|/c,\mathbf{x})\frac{x_1}{c|\mathbf{x}|} \quad (21)$$

Notice the similarity with the recursion relation. Using Equation (21), we obtain

$$\langle f_\alpha(t,\mathbf{x};C), \psi(t,\mathbf{x})\rangle = -\iiint_{\mathbb{R}^3}\int_{\mathbb{R}} g_{\mathbf{0}}(s,\mathbf{x};C)\partial_1[\psi(s\pm|\mathbf{x}|/c,\mathbf{x})]\,\mathrm{d}s\,\mathrm{d}^3\mathbf{x} \pm \iiint_{\mathbb{R}^3}\int_{\mathbb{R}} g_{\mathbf{0}}(s,\mathbf{x};C)\frac{x_1}{c|\mathbf{x}|}(\partial_0\psi)(s+|\mathbf{x}|/c,\mathbf{x})\,\mathrm{d}s\,\mathrm{d}^3\mathbf{x} \quad (22)$$

Since the integrands are continuous and have compact support, we can use Fubini's theorem and integrate successively over each space-time coordinate. We can, for example, start by integrating with respect to x_1 over $(-\infty,\infty)$. Using integration by parts, we get

$$= -\iint_{\mathbb{R}^2}\int_{\mathbb{R}} g_{\mathbf{0}}(s,\mathbf{x};C)\psi(s\pm|\mathbf{x}|/c,\mathbf{x})|_{x_1=-\infty}^{\infty}\mathrm{d}s\,\mathrm{d}x_2\,\mathrm{d}x_3 + \iiint_{\mathbb{R}^3}\int_{\mathbb{R}}(\partial_1 g_{\mathbf{0}})(s,\mathbf{x};C)\psi(s\pm|\mathbf{x}|/c,\mathbf{x})\,\mathrm{d}s\,\mathrm{d}^3\mathbf{x}$$
$$\pm\iiint_{\mathbb{R}^3} g_{\mathbf{0}}(s,\mathbf{x};C)\frac{x_1}{c|\mathbf{x}|}\psi(s\pm|\mathbf{x}|/c,\mathbf{x})|_{s=-\infty}^{\infty}\mathrm{d}^3\mathbf{x} \mp \iiint_{\mathbb{R}^3}\int_{\mathbb{R}}(\partial_0 g_{\mathbf{0}}(s,\mathbf{x};C))\frac{x_1}{c|\mathbf{x}|}\psi(s\pm|\mathbf{x}|/c,\mathbf{x})\,\mathrm{d}s\,\mathrm{d}^3\mathbf{x} \quad (23)$$

Since ψ has compact support, $\psi(s\pm|\mathbf{x}|/c,\mathbf{x})|_{x_1=-\infty}^{\infty} = 0$ for any fixed s, and $\psi(s\pm|\mathbf{x}|/c,\mathbf{x})|_{s=-\infty}^{\infty} = 0$ for any fixed $\mathbf{x}$. Hence

$$\langle f_\alpha(t,\mathbf{x};C), \psi(t,\mathbf{x})\rangle = \iiint_{\mathbb{R}^3}\int_{\mathbb{R}}\left[(\partial_1 g_{\mathbf{0}})(s,\mathbf{x};C) \mp (\partial_0 g_{\mathbf{0}})(s,\mathbf{x};C)\frac{x_1}{c|\mathbf{x}|}\right]\psi(s\pm|\mathbf{x}|/c,\mathbf{x})\,\mathrm{d}s\,\mathrm{d}^3\mathbf{x}$$
$$= \iiint_{\mathbb{R}^3}\int_{\mathbb{R}} g_\alpha(s,\mathbf{x};C)\psi(s\pm|\mathbf{x}|/c,\mathbf{x})\,\mathrm{d}s\,\mathrm{d}^3\mathbf{x} = \langle g_\alpha(t\mp|\mathbf{x}|/c,\mathbf{x};C), \psi(t,\mathbf{x})\rangle \quad (24)$$

by definition of g_1 and the change of coordinates in Equation (16). We have also rewritten the integral using the bracket notation $\langle\cdot,\cdot\rangle$. Notice that the proof is valid for any $\alpha \in \{(1,0,0),(0,1,0),(0,0,1)\}$, which shows the result for $|\alpha| = 1$.

We finish the proof by showing the recursion step: we assume that for $|\alpha| \geq 1$, $f_\alpha(t,\mathbf{x};C) = g_\alpha(t\mp|\mathbf{x}|/c,\mathbf{x};C)$ and want to show the result for the order $|\alpha|+1$. Given any multi-index α, all multi-indices of order $|\alpha|+1$ equal $\alpha+\mathbf{e}_i$ for some $i \in \{1,2,3\}$. Hence, we need to show that $f_{\alpha+\mathbf{e}_i}(t,\mathbf{x};C) = g_{\alpha+\mathbf{e}_i}(t\mp|\mathbf{x}|/c,\mathbf{x};C)$. Let us take a test function $\psi \in \mathscr{D}$ whose derivatives vanish at $\mathbf{x} = \mathbf{0}$ and notice that

$$\langle f_\alpha(t,\mathbf{x};C), (\partial_i\psi)(t,\mathbf{x})\rangle = \langle G(t,\mathbf{x}), \langle C(s)(D^\alpha\delta^3(\mathbf{y})), (\partial_i\psi)(t+s,\mathbf{x}+\mathbf{y})\rangle\rangle \quad (25)$$

(by definition of the convolution and f_α)

$$= (-1)^{|\alpha|}\langle G(t,\mathbf{x}), \langle C(s)\delta^3(\mathbf{y}), (D^{\alpha+\mathbf{e}_i}\psi)(t+s,\mathbf{x}+\mathbf{y})\rangle\rangle = (-1)^{|\alpha|}\left\langle G(t,\mathbf{x}), \int_{\mathbb{R}} C(s)(D^{\alpha+\mathbf{e}_i}\psi)(t+s,\mathbf{x})\,\mathrm{d}s\right\rangle \quad (26)$$

(by definition of the derivative of a distribution, then by Equation (9) of the main text)

$$= (-1)^{|\alpha|}\left\langle \frac{\pm 1}{4\pi}\frac{\delta(t\mp|\mathbf{x}|/c)}{|\mathbf{x}|}, \int_{\mathbb{R}} C(s)(D^{\alpha+\mathbf{e}_i}\psi)(t+s,\mathbf{x})\,\mathrm{d}s\right\rangle \quad (27)$$

(by definition of Green's distribution)

$$= (-1)^{|\alpha|}\iiint_{\mathbb{R}^3}\int_{\mathbb{R}} g_{\mathbf{0}}(s,\mathbf{x};C)(D^{\alpha+\mathbf{e}_i}\psi)(s\pm|\mathbf{x}|/c,\mathbf{x})\,\mathrm{d}s\,\mathrm{d}^3\mathbf{x} \quad (28)$$

by definition of $g_{\mathbf{0}}$. On the other hand, since $\partial_i \psi$ vanishes at $\mathbf{x} = \mathbf{0}$, we can compute

$$\langle f_\alpha(t,\mathbf{x};C),(\partial_i\psi)(s,\mathbf{x})\rangle = \iiint_{\mathbb{R}^3}\int_{\mathbb{R}} f_\alpha(t,\mathbf{x};C)(\partial_i\psi)(t,\mathbf{x})\,\mathrm{d}t\,\mathrm{d}^3\mathbf{x} = \iiint_{\mathbb{R}^3}\int_{\mathbb{R}} g_\alpha(t\mp|\mathbf{x}|/c,\mathbf{x};C)(\partial_i\psi)(t,\mathbf{x})\,\mathrm{d}t\,\mathrm{d}^3\mathbf{x} \tag{29}$$

(by the recursion assumption)

$$= \iiint_{\mathbb{R}^3}\int_{\mathbb{R}} g_\alpha(s,\mathbf{x};C)(\partial_i\psi)(s\pm|\mathbf{x}|/c,\mathbf{x})\,\mathrm{d}s\,\mathrm{d}^3\mathbf{x} \tag{30}$$

by making the inverse change of coordinates described in Equation (16). We will need this later.

Now, let us again use the convolution to find $f_{\alpha+\mathbf{e}_i}$:

$$\langle f_{\alpha+\mathbf{e}_i}(t,\mathbf{x};C),\psi(t,\mathbf{x})\rangle = (-1)^{|\alpha+\mathbf{e}_i|}\iiint_{\mathbb{R}^3}\int_{\mathbb{R}} g_{\mathbf{0}}(s,\mathbf{x};C)(D^{\alpha+\mathbf{e}_i}\psi)(s\pm|\mathbf{x}|/c,\mathbf{x})\,\mathrm{d}s\,\mathrm{d}^3\mathbf{x} \tag{31}$$

(by the exact same procedure that led to Equation (28))

$$= -(-1)^{|\alpha|}\iiint_{\mathbb{R}^3}\int_{\mathbb{R}} g_{\mathbf{0}}(s,\mathbf{x};C)(D^{\alpha}\partial_i\psi)(s\pm|\mathbf{x}|/c,\mathbf{x})\,\mathrm{d}s\,\mathrm{d}^3\mathbf{x} = -\langle f_\alpha(t,\mathbf{x};C),\partial_i\psi(t,\mathbf{x})\rangle \tag{32}$$

(again by the procedure that led to Equation (28))

$$= -\iiint_{\mathbb{R}^3}\int_{\mathbb{R}} g_\alpha(s,\mathbf{x};C)(\partial_i\psi)(s\pm|\mathbf{x}|/c,\mathbf{x})\,\mathrm{d}s\,\mathrm{d}^3\mathbf{x} \tag{33}$$

(by Equation (30))

$$= \iiint_{\mathbb{R}^3}\int_{\mathbb{R}} \left[\partial_i g_\alpha(s,\mathbf{x};C)\mp(\partial_0 g_\alpha)(s,\mathbf{x};C)\frac{x_i}{c|\mathbf{x}|}\right]\psi(s\pm|\mathbf{x}|/c,\mathbf{x})\,\mathrm{d}s\,\mathrm{d}^3\mathbf{x} \tag{34}$$

(by integration by parts and the compact support of ψ – recall Equation (21))

$$= \iiint_{\mathbb{R}^3}\int_{\mathbb{R}} \left[D^{\mathbf{e}_i} g_\alpha(s,\mathbf{x};C)\mp(\partial_0 g_\alpha)(s,\mathbf{x};C)\frac{\mathbf{x}^{\mathbf{e}_i}}{c|\mathbf{x}|}\right]\psi(s\pm|\mathbf{x}|/c,\mathbf{x})\,\mathrm{d}s\,\mathrm{d}^3\mathbf{x} \tag{35}$$

(by definition of the multi-index $\mathbf{e}_i$)

$$= \iiint_{\mathbb{R}^3}\int_{\mathbb{R}} g_{\alpha+\mathbf{e}_i}(s,\mathbf{x};C)\psi(s\pm|\mathbf{x}|/c,\mathbf{x})\,\mathrm{d}s\,\mathrm{d}^3\mathbf{x} = \iiint_{\mathbb{R}^3}\int_{\mathbb{R}} g_{\alpha+\mathbf{e}_i}(t\mp|\mathbf{x}|/c,\mathbf{x};C)\psi(t,\mathbf{x})\,\mathrm{d}t\,\mathrm{d}^3\mathbf{x} \tag{36}$$

(by definition of $g_{\alpha+\mathbf{e}_i}$, see the recursion relation, then by the change of coordinates described in Equation (16))

$$= \langle g_{\alpha+\mathbf{e}_i}(t\mp|\mathbf{x}|/c,\mathbf{x};C),\psi(t,\mathbf{x})\rangle \tag{37}$$

which shows the statement and concludes the proof. □

C Computation of the time-domain multipole moments

Computing the time-domain moments linked to the current density amounts to computing the integral

$$\int_{\mathbb{R}} x_1^{\alpha_1}\Pi\left(\frac{x_1-x_1^0}{\sigma_1}\right)\mathrm{d}x_1 = \left.\frac{x_1^{\alpha_1+1}}{\alpha_1+1}\right|_{x_1=x_1^0-\sigma_1/2}^{x_1=x_1^0+\sigma_1/2} \tag{38}$$

The charge density involves the second-order derivative with respect to x_1:

$$\partial_1^2\Pi\left(\frac{x_1-x_1^0}{\sigma_1}\right) = \partial_1\left[\delta(x_1-x_1^0+\sigma_1/2)-\delta(x_1-x_1^0-\sigma_1/2)\right] = \delta'(x_1-x_1^0+\sigma_1/2)-\delta'(x_1-x_1^0-\sigma_1/2) \tag{39}$$

where we have used the scaling property of the Dirac δ distribution. The moments give

$$\int_{\mathbb{R}}\left(\delta'(x_1-x_1^0+\sigma_1/2)-\delta'(x_1-x_1^0-\sigma_1/2)\right)x_1^{\alpha_1}\,\mathrm{d}x_1 = \left.\alpha_1 x_1^{\alpha_1-1}\right|_{x_1=x_1^0-\sigma_1/2}^{x_1=x_1^0+\sigma_1/2} \tag{40}$$

if $\alpha_1 > 0$, and zero otherwise.

References

1. van Dijk, G. *Distribution Theory: Convolution, Fourier Transform, and Laplace Transform* (De Gruyter, 2013).
2. Lechner, K. *Classical Electrodynamics: A Modern Perspective* (Springer, Cham, Switzerland, 2018).
3. Mitrea, D. *Distributions, Partial Differential Equations, and Harmonic Analysis*. Universitext (Springer-Verlag, New York, 2013).
